# Predictors of Urinary Arsenic Levels among Postmenopausal Danish Women

**DOI:** 10.3390/ijerph15071340

**Published:** 2018-06-26

**Authors:** Nina Roswall, Ulla A. Hvidtfeldt, James Harrington, Keith E. Levine, Mette Sørensen, Anne Tjønneland, Jaymie R. Meliker, Ole Raaschou-Nielsen

**Affiliations:** 1 Danish Cancer Society Research Center, Strandboulevarden 49, 2100 Copenhagen, Denmark; roswall@cancer.dk (N.R.); ullah@cancer.dk (U.A.H.); mettes@cancer.dk (M.S.); annet@cancer.dk (A.T.); 2 Analytical Sciences Division, Research Triangle Institute, 3040 East Cornwallis Road, PO Box 12194, Research Triangle Park, NC 27709-2194, USA; jharrington@rti.org (J.H.); levine@rti.org (K.E.L.); 3 Department of Natural Science and Environment, Roskilde University, Universitetsvej 1, 4000 Roskilde, Denmark; 4 Program in Public Health, Department of Family, Population, and Preventive Medicine, Stony Brook University, 101 Nicolls Road, Stony Brook, NY 11794, USA; Jaymie.meliker@stonybrookmedicine.edu; 5 Department of Environmental Science, Aarhus University, Frederiksborgvej 399, 4000 Roskilde, Denmark

**Keywords:** arsenic, determinants, predictors, diet, lifestyle, demography

## Abstract

Arsenic is a risk factor for several noncommunicable diseases, even at low doses. Urinary arsenic (UAs) concentration is a good biomarker for internal dose, and demographic, dietary, and lifestyle factors are proposed predictors in nonoccupationally exposed populations. However, most predictor studies are limited in terms of size and number of predictors. We investigated demographic, dietary, and lifestyle determinants of UAs concentrations in 744 postmenopausal Danish women who had UAs measurements and questionnaire data on potential predictors. UAs concentrations were determined using mass spectrometry (ICP-MS), and determinants of the concentration were investigated using univariate and multivariate regression models. We used a forward selection procedure for model optimization. In all models, fish, alcohol, and poultry intake were associated with higher UAs concentration, and tap water, fruit, potato, and dairy intake with lower concentration. A forward regression model explained 35% (*R*^2^) of the variation in concentrations. Age, smoking, education, and area of residence did not predict concentration. The results were relatively robust across sensitivity analyses. The study suggested that UAs concentration in postmenopausal women was primarily determined by dietary factors, with fish consumption showing the strongest direct association. However, the majority of variation in UAs concentration in this study population is still unexplained.

## 1. Introduction

Arsenic is a ubiquitous element in the environment, where it occurs in both organic and inorganic forms. Inorganic forms are primarily present in water, rendering drinking water the primary route of human exposure to these species. In contrast, exposure to organic arsenicals primarily occurs through food ingestion [1]. Although the primary route of total arsenic exposure globally is food ingestion, some areas (e.g., Bangladesh, Taiwan, and Chile) have elevated inorganic arsenic levels in the drinking water, rendering this the primary regional exposure pathway [2]. Finally, some occupations, including nonferrous smelting, arsenic production, wood preservation, glass manufacturing, production and application of arsenic-based pesticides, and electronics, elevate the risk of arsenic inhalation [2], which also entail higher urinary arsenic (UAs) concentrations and adversely affect health, similarly to arsenic ingestion [3,4].

Concern about the role of arsenic exposure in relation to global public health has risen as some forms of organoarsenic compounds and inorganic arsenic have been classified as “carcinogenic to humans” by the International Agency for Research on Cancer and have been shown to induce oxidative stress and DNA damage [2]. Furthermore, inorganic arsenic exposure has also been found to be a risk factor for hypertension, cardiovascular disease, and diabetes, even in populations with low levels of exposure [5,6,7,8]. 

The European Food Safety Authority has detected low levels of arsenic in almost all food items (typical concentrations less than 0.25 mg/kg). Factors influencing total arsenic in the diet include food type, growing conditions, food-processing techniques, and the arsenic concentration in cooking water [1]. Many previous studies on dietary predictors of UAs in populations with low exposure through drinking water have focused on fish and shellfish consumption [9,10,11,12,13], as they are generally considered the foods with the highest total arsenic levels [1]. However, several other dietary factors have been proposed to be predictors, including rice and poultry [1,14,15,16], as have also demographic and behavioral factors, such as age, gender, and body mass index [11,13,17,18]. However, only a few studies have explored predictors of UAs concentrations in low-level exposure populations, employing both a broad number of potential determinants and using well-validated information on UAs concentrations. From a public health perspective, a deeper understanding of the predictors of UAs concentrations will facilitate the prevention of chronic, noncommunicable diseases induced by arsenic exposure.

The purpose of the present study was to identify the demographic, dietary, and lifestyle factors that are predictors of urinary arsenic levels among postmenopausal women in the Danish Diet, Cancer and Health (DCH) cohort.

## 2. Materials and Methods

From 1 December 1993 through 31 May 1997, a total of 57,053 individuals (29,875 women and 27,178 men), aged 50–64 years, born in Denmark, and with no previous cancer diagnosis in the Danish Cancer Registry, were enrolled in the prospective DCH cohort [19]. At enrolment, each participant gave a urine sample and completed a self-administered, interviewer-checked, 192-item semiquantitative food frequency questionnaire, as well as a comprehensive lifestyle questionnaire including information on smoking, occupational history, and health status. Anthropometric measures were collected by trained personnel. Among the 29,875 women, 338 had a cancer diagnosis before baseline, one had an unknown date of cancer diagnosis, and 157 did not provide a urinary sample, and were thus excluded. Among the remaining 29,379 women, 900 out of 1121 eligible postmenopausal breast cancer cases and a comparison group of 898 postmenopausal women were selected for a case-cohort study on cadmium and breast cancer, as described in detail previously [20]. For 745 randomly selected women among these 1798 women, we measured not only cadmium but also UAs levels, and these women were included in the present study. Of the 745 women, 388 developed breast cancer between 4 years after the baseline visit and the end of 2012, and 357 women did not develop breast cancer between baseline and 2012. In order to account for this, case status is included as a covariate in the analyses.

The study was conducted in accordance with the Declaration of Helsinki, and the protocol was approved by the regional research ethic committee for Copenhagen and Frederiksberg (Approval no.: (KF) 01-345/93). Written informed consent was obtained from all study participants upon enrolment into the cohort. The present analysis was carried out without contact to the cohort members or their families.

Spot urine samples from each of the 745 women were shipped on dry ice and analyzed at Research Triangle Institute’s Analytical Science Laboratory (Research Triangle Park, NC, USA). Arsenic was measured in the urine samples, which were collected in transparent polypropylene cups (USON Plast, Svendborg, Denmark) and stored in transparent 1 ml polypropylene cryotubes (NUNC, Roskilde, Denmark). UAs concentrations were determined using a Thermo X-Series 2 (Bremen, Germany) inductively coupled plasma mass spectrometer (ICP-MS) following digestion in the presence of high-purity acids and oxidants in a Class 100 clean hood to prevent contamination from environmental sources. A detailed description of the ICP-MS procedure is described elsewhere [20]. A detailed description of sample preparation procedures, analytical method, and quality control measures is provided in the supplementary methods section. Urinary creatinine concentration was quantified using a Cayman Chemicals Creatinine Assay Kit No. 500701 (Cayman Chemicals, Ann Arbor, MI, USA) with UV-VIS measurement at 500 nm employing a Beckman Coulter DU800 UV/VIS Spectrometer (Beckman Instruments Inc., Brea, CA, USA). 

The distribution of the measured values of UAs concentration was right-skewed, and we therefore log-transformed the concentration prior to statistical analyses in order to obtain a normal distribution.

Association between log-transformed UAs levels and baseline study characteristics were analyzed in linear regression analyses, including univariate regression, multivariate regression including all potential predictors, and finally using an unsupervised forward regression model to determine a reduced model. The model optimization procedure added variables at each step that provided the greatest value of the adjusted *R*^2^ statistic, stopping at the step where the significance level corresponding to the addition of a predictor was greater than 0.2. 

The predictor variables used in the model selection procedure were age (years), BMI (kg/m^2^), alcohol intake (g/day), abstainer (yes/no), smoking status (never, former, current), years of school attendance (<8, 8–10, >10 years), area of residence (Copenhagen, Aarhus), total fish intake (g/d), total prawn intake (g/day), red meat (g/day), poultry (g/day), tap water (L/d), all vegetables (g/day), cruciferous vegetables (g/day), leafy vegetables (g/day), all fruits (g/day), all potatoes (g/day), rice (g/day), cereals (g/day), dairy products (g/day), ever worked for >1 year in the glass industry (yes/no), ever worked for >1 year in the wood industry (yes/no), estimated arsenic in drinking water (µg/L) (calculated as described in [21]), later development of breast cancer (yes/no), and urinary creatinine concentration (g/L). Potential predictors included in the selection procedure were selected based on a priori knowledge about determinants for arsenic concentrations. 

In order to exclude the possibility that included variables would be selected because of association with creatinine rather than with UAs, we constructed models using UAs as the dependent variable and added urinary creatinine as a predictor instead of using it to normalize the UAs concentration, as recommended by Barr et al. [22]. This was done because it allows appropriate adjustment for urinary creatinine while permitting the statistical effects of the remaining covariates in the model to be independent of the effects of the creatinine concentration [22]. As a sensitivity analysis, we also conducted the analyses with creatinine-normalized arsenic concentrations, instead of including creatinine as a covariate. 

As further sensitivity analyses, we calculated all models for women in the lowest quartile of fish consumption and for women who did not develop breast cancer in order to investigate the robustness of our findings.

The procedure PROC GLM was used for regression analyses. The procedure PROC GLMSELECT was used for the model optimization procedure, specifying the option “selection = forward (select = ADJRSQ stop = SL SLE = 0.2)”. All analyses were performed in SAS version 9.4 (SAS Institute, Cary, NC, USA).

Data from the Diet, Cancer and Health cohort is not publicly available due to Danish legislation concerning protection of personal data. Admission to accessing data is based on application to the principal investigators Anne Tjønneland and Kim Overvad, through whom the application will be distributed to the steering committee of the Diet, Cancer and Health cohort, who will then process the application and return to the applicant with a final decision regarding access to data. Also, acquisition of data is only allowed after permission to handle data has been obtained from the Danish Data Protection Agency: http://www.datatilsynet.dk/english. The e-mail address for Dr. Anne Tjønneland is: annet@cancer.dk. The e-mail address for Dr. Kim Overvad is: ko@ph.au.dk.

## 3. Results

Among the 745 women initially included in the study, one woman lacked information on covariates and was excluded from the analytical study population, which then consisted of 744 postmenopausal women. 

The distribution of lifestyle factors, daily intake of major dietary groups, occupational history, arsenic consumption through drinking water, and urinary creatinine concentrations in the analyzed samples are presented in Table 1. The study participants had a median age of 57 years and a median BMI of 25 kg/m^2^. The majority of the participants lived in the Copenhagen area, and the dominant dietary groups were dairy products, fruits, vegetables, and cereal. Very few participants had worked in industries with potential arsenic exposure. 

Table 2 presents the distribution of UAs concentrations in the study population, both with and without adjustment for creatinine. There was wide variation in urinary arsenic concentrations among participants. The arithmetic and geometric mean UAs concentrations were 37.87 and 16.34 µg/L, respectively. The corresponding values after creatinine normalization were 63.77 and 33.77 µg/g, respectively. The correlation (R_Spearman_) between UAs and urinary creatinine was 0.59.

Results of the univariate and multivariate regression model are shown in Table 3. In the univariate regression models, alcohol, fish, prawn, and poultry intake as well as creatinine were significantly positively associated with log-transformed UAs concentrations, and tap water and dairy products were negatively associated with UAs concentrations. In the multivariate model, including all variables, alcohol, fish, and poultry intake as well as creatinine remained significantly positively associated with UAs concentration, and tap water and dairy remained negatively associated, but fruits and potatoes also showed significant inverse associations with UAs.

The unsupervised forward model selection procedure selected 11 out of 22 possible predictor variables (Table 4). The final model explained 34.75% of the variation in UAs concentration (adjusted *R*^2^ value). This model included (in order of decreasing adjusted *R*^2^ value magnitude): Urinary creatinine levels, fish consumption, dairy product consumption, fruit consumption, alcohol consumption, potato consumption, tap water consumption, poultry consumption, working in the glass industry, vegetable consumption, BMI, and leafy vegetable consumption.

As sensitivity analyses, we repeated the above analyses for log-transformed, creatinine-normalized arsenic concentrations. The results of this were highly similar to the non-normalized analyses, with alcohol and fish being the primary predictors of higher UAs levels and fruits, potatoes, and dairy being the primary predictors of lower UAs levels. In contrast to the non-normalized model, however, poultry intake was only nonsignificantly associated with higher UAs concentrations (*p* = 0.12), whereas estimated arsenic in drinking water was now significantly directly associated with UAs concentration (*p* = 0.04). We also repeated the analyses among women who did not develop breast cancer only. The results of this were highly similar to the main analysis, with the only difference in the multivariate analysis that alcohol and poultry consumption were now only borderline significantly associated with higher UAs levels (*p* = 0.07), and tap water was no longer a predictor (*p* = 0.38). 

As a final sensitivity analysis, we repeated the regression analyses for women with the lowest quartile of fish consumption (≤23.9 g/day), in order to investigate whether the predictors of UAs differed in this group (Table S1). The non-normalized median UAs concentration in this group was 13.3 and the arithmetic mean was 30.4 µg/L. However, in the multivariate models, fish consumption, now along with prawn intake, were still the primary predictors of higher UAs concentrations after creatinine concentration, and alcohol and case status were borderline significantly associated with higher concentrations. In the forward regression model, fruit, dairy, and potato consumption were also included as predictors of lower UAs concentrations (Table S2). 

## 4. Discussion

The current study suggests a significant association between fish, alcohol, and poultry consumption and higher UAs concentration as well as a significant inverse association with tap water, fruit, potato, and dairy. Model optimization using a forward regression model included also leafy vegetable intake and working in the glass industry as predictors of a higher UAs concentration, and BMI and total vegetable intake as determinants of a lower concentration, albeit the effect was nonsignificant. The included variables were able to explain only 35% of the variation in UAs concentrations. Fish consumption was the dietary predictor that explained most of the variation. We found no association with age, smoking, education, and area of residence. The results were relatively robust across sensitivity analyses.

### 4.1. Study Strengths and Limitations

A prominent study strength was the high-quality UAs measurements. UAs is generally considered the most reliable indicator of arsenic exposure, since it is the primary route of excretion for most arsenic species [1,23,24,25]. This is also the case when using spot urine samples [26], as in the present study, since UAs concentration is stable throughout the day [27]. About 75% of total UAs consists of organic forms [23] and total UAs levels have been accepted as a good biomarker of recent exposure. The study had a reasonable sample size for studying potential predictors of UAs levels. The information on covariates (lifestyle, occupation, demographics, and diet) were also of high quality, having been collected through detailed, interviewer-checked questionnaires. The dietary variables, a main component of the current analyses, have been validated in relation to weighed diet records, producing good agreement [28].

One potential study limitation was that only a single urine sample was analyzed from each subject, which suffers from temporal variability of the arsenic biomarker. However, a study in 196 arsenic-exposed individuals demonstrated a reasonable reproducibility of total UAs concentrations over 2 years [29]. A major limitation of the current study was that we were not able to differentiate between the organic arsenic forms and the more toxic inorganic forms, as speciation analysis was not included in the study design. Such an analysis could be integrated. In a future study, it could be relevant to refine the laboratory analysis for arsenic by chemical speciation to provide additional information on exposure sources and toxicology. Total UAs can be a useful marker of exposure to inorganic arsenic if ingestion of marine foods is limited, since seafood intake can produce a rapid and short-term increase in organic and thereby total UAs levels [30]. The current study had a median fish intake of 36 g/day and prawn intake of 1.7 g/day and included no restriction of intake of these in the days prior to sample collection. Therefore, as discussed in more detail below, the concentrations found in our study may reflect recent organic arsenic consumption via seafood, especially since the arsenic levels in Danish drinking water are relatively low [31,32]. However, a sensitivity analysis in women with a generally low fish consumption produced highly similar results to the main analysis. Another study limitation is potential exposure misclassification of the included predictors, as all dietary items were self-reported and as arsenic concentration in tap water was estimated based on water utilities outlets and not measured at the consumers tap [8,21].

### 4.2. Geographical Variation in Urinary Arsenic Concentrations

In order to facilitate comparison with other studies on general adult populations, which are not occupationally exposed or reside in highly contaminated areas, the distribution of UAs concentrations is presented with arithmetic and geometric means, as well as with and without creatinine normalization (Table 2). The Canadian Health Measures Survey found geometric mean values for women aged 60–79 years of 1.09 µg/L (95% CI 0.86–1.39), but did not report fish consumption [33]. The US NHANES study [23] reported a geometric mean among adults (≥20 years) of 24.6 µg/L among those who had consumed seafood in the past 24 h and 7.3 µg/L among those that did not [9]. In the French National Nutrition and Health Survey, where participants were not allowed to consume fish or seafood for 3 days before sample collection, the geometric mean among adult women (18+ years) was 12.06 µg/g creatinine, 95% CI: 11.39–12.78. The majority of participants normally consumed fish/shellfish once a week to twice a month [17]. In the German Environmental Surveys, the geometric mean was 5.64 µg/L for adults who consumed fish up to three times per month and 7.46 µg/L for those with fish consumption >3 times/month [34]. Finally, the only previous Danish study found a median concentration of 11.24 µg/L in women aged 40–70 years. However, there was no association with fish consumption, the frequency of which was not reported [35]. Despite not including completely comparable groups, these studies generally found lower UAs concentrations than we do in the present study. With the limited information on fish consumption in these studies, it is not possible to ascribe the difference to a higher fish consumption in our study. However, a South Korean study found a geometric mean UAs of 93 µg/L in females, which they attributed to very high seafood intake (53.6 g/day in females) [13], and a study in adult Japanese men found a median concentration of UAs of 141.3 µg/L, with a reported daily consumption of fish and seafood products of 187 g [36], which are considerably higher intakes and UAs concentrations than in our study. Also, several studies of adult populations living in areas known to have very high arsenic concentrations in local water sources have found much higher UAs concentrations in, e.g., Chile [37], Inner Mongolia [38], Mexico [39], Bangladesh [40], and Romania [41].

Several studies have also suggested large within-country variation in arsenic concentration, e.g., in Germany [34], the United States [23], France [42], and South Korea, after adjustment for seafood intake [13]. The variation most likely reflects local dietary patterns or local environmental contamination of soil and drinking water. We did not observe significant regional differences in this study, which included women from both the Copenhagen and Aarhus areas, even though regional variations in arsenic concentrations in both drinking water and soil have been found in Denmark [43,44,45]. There was limited regional difference in fish consumption between the two cities, with median intakes of 34.0 and 36.4 g/day in Aarhus and Copenhagen, respectively.

### 4.3. Dietary Predictors

The positive association with fish consumption in our study corresponds well with the knowledge that fish has very high arsenic levels [1] and with reports of increased urinary arsenic concentrations found in an intervention study with seafood [46]. Furthermore, several studies have found fish and seafood consumption to predict UAs concentrations [9,10,11,12,13,15,16,47,48]. The finding of a significant association with poultry corresponds to a similar association in the NHANES study [14,16], which may be explained by the use of arsenic-based drugs in poultry production, such as roxarsone, at time of sample collection [14]. While associated with a nonsignificant difference in UAs in the multivariate model only, leafy vegetables were included in the forward selection model as a predictor. In the NHANES study, vegetables were considered as an overall category only, and this was marginally inversely (0.05 < *p* ≤ 0.15) associated with arsenic metabolites [16]. In our study, the overall group of all vegetables was also inversely associated with UAs concentrations, albeit nonsignificantly, as was the subgroup of cruciferous vegetables. It has been suggested that leafy vegetables grown in arsenic-contaminated soil reach higher arsenic concentrations compared to other types of vegetables [49,50], which may explain our findings. We do not, however, have any information on where the leafy vegetables consumed in our study were grown. We also found a direct association between alcohol and UAs, which corresponds with the results of some [15,17,51] but not all previous studies [11].

Dairy product consumption was inversely associated with UAs. This is in accordance with previous studies in adults on milk or dairy products [13,16,48]. The inverse association with fruit consumption was also seen in a Spanish study [48], whereas the NHANES study and a Korean study both found a direct association [15,16]. In Denmark, fruits are estimated to contribute very little to the total arsenic intake [52]. These inverse associations may be explained by the fact that these products are low in arsenic and that women who consume large amounts of these food groups consume relatively less of other food groups with higher arsenic concentrations. We also observed an inverse association with potato consumption. Few studies have investigated this relationship, and the above-mentioned Korean and Spanish studies found no association [15,48]. Neither study reported daily potato consumption, but in the present study, this was relatively high (median 120 g/day). The arsenic concentration in foods vary based on growing conditions (type of soil, water, geochemical activity, use of arsenic-based pesticides), processing techniques [53,54], and arsenic concentration in the cooking water [1], and this could potentially explain the discrepancies between these studies from different countries. We did not find an association with rice consumption, as some other studies have [15,16]. These studies did not report on the amount of rice consumed, but in the present study, it was relatively low (10 g/day), so we may not have been able to detect an association.

### 4.4. Water and Arsenic Concentration in Water

We did not find arsenic in drinking water to be a predictor of UAs concentration. This is in contrast to other studies in populations with low arsenic exposure in water [10,55]. The lack of association could possibly be explained by misclassification of arsenic exposure, as this was based on measurements at water utilities outlets, as described in [8,21], and we had no information on arsenic concentrations at the consumers tap, which could differ due to arsenic dissolving back into the water from the pipes. However, there is generally a low-level release of arsenic to drinking water in Denmark [31], as also reflected in the low water concentration of arsenic in the present study, which was well below the current provisional guidelines from the World Health Organization of 10 µg/L [56]. There was also a low variation in exposure, which may render it impossible to see an effect over such a small exposure spectrum. We did, however, find an inverse association between total tap water consumption and UAs concentration. This may be explained by the low tap water arsenic concentrations in Denmark: The Danish drinking water standard at the consumers tap is a maximum of 10 µg/L [32], and with such a low concentration, water consumption may dilute the arsenic concentration in urine achieved from other sources. Two American studies found a direct association between tap water consumption and UAs [11,18], whereas a third American study and an Australian study did not [12,16]. The American study finding no association ascribed this to the low concentration of arsenic in US drinking water, which averages 2 µg/L [16]. The two American studies finding an association reported tap water concentrations ranging from <3 (i.e., the lower limit of detection) to 1200 µg/L and from <3 to 1850 µg/L, respectively, in their study populations [11,18]. Thus, at least some of their study populations were exposed to much higher drinking water arsenic concentrations than in our study.

### 4.5. Demographic and Lifestyle Factors

We found no association with BMI in univariate or multivariate analyses, but in the forward selection model, it was included as a predictor. Previous studies on BMI and UAs concentrations have shown diverging results [10,17,18]. Some studies have suggested that smoking is a predictor for UAs [11,18], but the present study, as well as some other studies, did not find an association [10,13]. Finally, a number of studies have suggested that age is a predictor for UAs levels [11,13,17,18,37,55]. This was not the case in our study, but the relatively narrow age group included here (50–64 years) may explain this discrepancy.

### 4.6. Occupational Arsenic Exposure

We found no significant association with working in the wood or glass industry in neither the univariate nor the multivariate model. Working in the glass industry was included in the reduced model. However, as only one woman worked in the glass industry, and as we have no information on what specific position she held, we do not find it reasonable to conclude anything based on such small numbers.

### 4.7. Generalizability of the Study Findings

Generalizability of our results should be exerted with caution. Several studies have suggested that age and gender are predictors of UAs concentrations [11,13,17,18,37,55], and as the present study includes a relatively homogeneous population of postmenopausal women, the results may not be generalized to other groups, e.g., men or younger women. Furthermore, the results of the present study identify predictors for UAs concentrations in a population with low arsenic concentrations in drinking water. In populations with high exposure through drinking water, this is a strong predictor of UAs concentrations [40,41]. We conducted three sensitivity analyses in the present study: recalculating the analyses with creatinine normalization of the arsenic concentration, calculating the analyses among women with low fish consumption only, and among women who did not develop breast cancer only, and our findings were relatively homogeneous across all models.

## 5. Conclusions

In conclusion, the results of the present study suggest that the UAs concentration in a population of postmenopausal Danish women with low exposure to arsenic through drinking water was primarily determined by dietary factors. Our models explained 35% of the variation, with fish consumption being the strongest dietary determinant. This finding was robust across sensitivity analyses. The majority of variation in UAs concentration in the study population is thus unexplained in the present study, which may be a result of a lack of inclusion of unknown predictors or by exposure misclassification of the included variables in our models.

## Figures and Tables

**Table 1:** Baseline characteristics of the study population (*N* = 744).

Baseline Characteristics	Distribution *N* (%)	Median (5th–95th Percentile)
**Lifestyle factors**		
Age (years)		57 (51–64)
BMI (kg/m^2^)		25 (20–33)
Abstainers	28 (3.8)	
Alcohol intake (g/day) ^a^		10 (0.7–46)
Smoking status		
- Never	315 (42)	
- Former	161 (22)	
- Current	268 (36)	
Years of school attendance		
- Low (<8 years)	276 (37)	
- Medium (8–10 years)	348 (47)	
- High (>10 years)	120 (16)	
Area of residence		
- Copenhagen	530 (71)	
- Aarhus	214 (29)	
**Dietary groups** (g/day unless otherwise stated)		
Fish ^b^		36 (11–86)
Prawns		1.7 (0.0–7.2)
Red meat ^c^		65 (27–131)
Poultry ^d^		15 (3–53)
Tap water (L/day) ^e^		2.0 (1.0–3.2)
All vegetables, incl. juices		167 (49–400)
Cruciferous vegetables		11 (1.2–40)
Leafy vegetables		7 (0.7–36)
All fruits, incl. juices		200 (40–553)
Potatoes		120 (33–306)
Rice		10 (2–49)
Cereals		167 (79–285)
Dairy products ^f^		304 (50–984)
**Occupation ^g^**		
Glass industry	1 (0.1)	
Wood industry	3 (0.4)	
**Arsenic in drinking-water** (µg/L)		0.57 (0.43–2.11)
**Urinary creatinine concentration** (g/L)		0.53 (0.12–1.81)

^a^ Among drinkers; ^b^ Sum of fresh and processed fish; ^c^ Beef, veal, pork, and lamb (including processed meat and offal); ^d^ Chicken and turkey; ^e^ Sum of tap water, coffee, tea, and fruit syrup diluted with tap water; ^f^ Milk, cheese, cream, yogurt, ice cream, and other cultured milk products; ^g^ Ever worked in the industry more than 1 year.

**Table 2:** Urinary arsenic concentrations in the study population (*N* = 744), with and without creatinine normalization.

	Urinary Arsenic Level (µg/L)	Urinary Arsenic Level, Creatinine Normalized (µg/g)
**Arithmetic mean (5; 95 percentile)**	37.87 (1.74; 155.87)	63.77 (6.32; 195.51)
**Geometric mean (95% confidence limit)**	16.34 (14.84; 18.00)	33.77 (31.23; 36.51)
**Range**	0.36; 641.41	0.74; 636.30
**Median**	17.31	32.62

**Table 3:** Association between log_10_-transformed urinary arsenic levels and baseline study characteristics. Results of univariate and multivariate regression models.

Study Variables	*N*	Univariate Regression Model			Multivariate Regression Model *			*R* ^2^
		Difference (ng/mL)	95% CI	*p* Value	Difference (ng/mL)	95% CI	*p* Value	
Age (years)	744	−0.003	−0.01; 0.01	0.58	−0.001	−0.01; 0.008	0.79	0.36
BMI (kg/m^2^)	744	0.003	−0.01; 0.01	0.55	−0.04	−0.01; 0.008	0.34	
Alcohol intake (g/day) ^a^	716	0.006	0.003; 0.008	<0.001	0.003	0.001; 0.006	0.005	
Smoking status								
- Never	315	Ref	-	-	Ref	-	-	
- Former	162	−0.03	−0.14; 0.08	0.55	0.008	−0.09; 0.10	0.87	
- Current	268	0.04	−0.05; 0.14	0.39	0.04	−0.04; 0.13	0.34	
Years of school attendance								
- Low (<8 years)	276	Ref	-	-	Ref	-	-	
- Medium (8–10 years)	348	0.06	−0.04; 0.14	0.23	0.02	−0.06; 0.10	0.61	
- High (>10 years)	120	0.03	−0.10; 0.15	0.64	0.02	−0.09; 0.13	0.67	
Area of residence								
- Copenhagen	530	Ref	-	-	Ref	-	-	
- Aarhus	214	−0.04	−0.13; 0.06	0.43	−0.03	−0.11; 0.06	0.58	
Fish, g/day ^b^	744	0.005	0.003; 0.006	<0.001	0.006	0.005; 0.008	<0.001	
Prawns, g/day	744	0.02	0.008; 0.03	0.0005	0.004	−0.006; 0.01	0.44	
Red meat, g/day ^c^	744	0.0006	−0.0007; 0.002	0.36	−0.0007	−0.002; 0.0004	0.21	
Poultry, g/day ^d^	744	0.002	0.00001; 0.005	0.05	0.002	0.0001; 0.004	0.04	
Tap water, L/day ^e^	744	−0.0001	−0.0002; −0.0005	0.0002	−0.00006	−0.0001; −0.000003	0.04	
All vegetables, incl. juices, g/day	744	−0.0003	−0.0006; 0.0001	0.17	−0.0004	−0.0009; 0.00006	0.09	
Cruciferous vegetables, g/day	744	−0.0008	−0.004; 0.002	0.62	−0.0008	−0.004; 0.002	0.60	
Leafy vegetables, g/day	744	0.0003	−0.003; 0.004	0.89	0.002	−0.001; 0.006	0.23	
All fruits, incl. juices, g/day	744	−0.0002	−0.0004; 0.00007	0.17	−0.0003	−0.0005; −0.00009	0.006	
Potatoes, g/day	744	−0.0004	−0.0009; 0.00003	0.07	−0.0005	−0.0009; −0.00003	0.04	
Rice, g/day	744	0.002	−0.0009; 0.005	0.17	0.0001	−0.002; 0.003	0.89	
Cereals, g/day	744	−0.0001	−0.0008; 0.0005	0.70	0.00005	−0.0006; 0.0007	0.88	
Dairy products, g/day ^f^	744	−0.0002	−0.0004; −0.00009	0.0008	−0.0001	−0.0003; −0.00007	0.002	
Arsenic in drinking-water, µg/L	744	−0.008	−0.04; 0.02	0.60	0.02	−0.007; 0.05	0.14	
Wood industry ^g^								
- No	741	Ref			Ref			
- Yes	3	0.02	−0.64; 0.68	0.96	−0.06	−0.60; 0.48	0.83	
Glass industry ^g^								
- No	743	Ref			Ref			
- Yes	1	0.79	−0.36; 1.93	0.18	0.85	−0.08; 1.79	0.07	
Creatinine	744	0.52	0.45; 0.58	<0.0001	0.51	0.45; 0.58	<0.0001	
Case status								
- Case	387	Ref			Ref			
- Noncase	357	−0.002	−0.09; 0.08	0.97	−0.01	−0.08; 0.06	0.75	

^a^ Among drinkers; ^b^ Sum of fresh and processed fish; ^c^ Beef, veal, pork, and lamb (including processed meat and offal); ^d^ Chicken and turkey; ^e^ Sum of tap water, coffee, tea, and fruit syrup diluted with tap water; ^f^ Milk, cheese, cream, yogurt, ice cream, and other cultured milk products; ^g^ Ever worked in the industry more than 1 year; * Later development of breast cancer (case status) as well as urinary creatinine concentration included as adjustment factors.

**Table 4:** Association between log_10_-transformed urinary arsenic levels and baseline study characteristics. Results of forward regression model.

Study Variables	Difference (ng/mL)	*p* Value	Adjusted *R*^2^
Intercept	1.0297	-	0.0000
Creatinine	0.5118	<0.0001	0.2388
Fish, g/day ^a^	0.007	<0.0001	0.2925
Dairy products, g/day ^b^	−0.0002	<0.0001	0.3099
All fruits, incl. juices, g/day	−0.0003	0.0001	0.3223
Alcohol intake (g/day) ^c^	0.003	0.0012	0.3309
Potatoes, g/day	−0.0005	0.0022	0.3385
Tap water, L/day ^d^	−0.00005	0.0525	0.3410
Poultry, g/day ^e^	0.002	0.0735	0.3429
Glass Industry ^f^			
- No	Ref.		
- Yes	0.83	0.0918	0.3446
All vegetables, incl. juices, g/day	−0.0004	0.1295	0.3458
BMI (kg/m^2^)	−0.006	0.1278	0.3469
Leafy vegetables, g/day	0.003	0.1954	0.3475

^a^ Sum of fresh and processed fish; ^b^ Milk, cheese, cream, yogurt, ice cream, and other cultured milk products; ^c^ Among drinkers; ^d^ Sum of tap water, coffee, tea, and fruit syrup diluted with tap water; ^e^ Chicken and turkey; ^f^ Ever worked in the industry more than 1 year.

## Supplementary Materials

The following are available online at http://www.mdpi.com/1660-4601/15/7/1340/s1, Additional details on urinary arsenic analysis, Table S1: Association between log_10_-transformed urinary arsenic levels and baseline study characteristics. Results of univariate and multivariate regression models. Analyses among women in the lowest quartile of fish consumption (≤23.9 g/day) only, Table S2: Association between log_10_-transformed urinary arsenic levels and baseline study characteristics. Results of forward regression model among women in the lowest quartile of fish consumption (≤23.9 g/day) only.
