# Supplementary material for: Predictors of Urinary Arsenic Levels among Postmenopausal Danish Women
Source: Int J Environ Res Public Health. 2018 Jun 26;15(7):1340. doi: 10.3390/ijerph15071340 (PMC6068487; doi:10.3390/ijerph15071340)
Supplement: Supplementary file 1 [file ijerph-15-01340-s001.pdf]

Table s1. Association between log_{10}-transformed urinary arsenic levels and baseline study characteristics. Results of univariate and multivariate regression models. Analyses among women in the lowest quartile of fish-consumption (<= 23.9 g/day) only.

Study variables	N	Univariate regression model			Multivariate regression model*			
		Difference (ng/mL)	95% CI	P value	Difference (ng/mL)	95% CI	P value	R^2
Age (yrs)	186	-0.01	-0.03; 0.01	0.31	0.002	-0.42; 1.97	0.20	
BMI (kg/m^2)	186	-0.003	-0.02; 0.02	0.77	-0.008	-0.02; 0.02	0.85	
Alcohol intake (g/day)[a]	170	0.007	0.002; 0.01	0.01	0.005	-0.0002; 0.01	0.06	
Smoking status								
- *Never*	73	Ref	-	-	Ref	-	-	
- *Former*	35	-0.13	-0.37; 0.12	0.31	-0.01	0.32; 0.24	0.33	
- *Current*	78	0.04	-0.15; 0.24	0.67	0.06	-0.11; 0.24	0.76	
Years of school attendance								
- *Low (<8 yrs)*	71	Ref	-	-	Ref	-	-	
- *Medium (8-10 yrs)*	83	0.005	-0.19; 0.20	0.96	-0.09	-0.27; 0.08	0.29	
- *High (>10 yrs)*	32	-0.03	-0.29; 0.22	0.80	-0.09	-0.33; 0.15	0.48	
Area of residence								
- *Copenhagen*	134	Ref	-	-	Ref	-	-	
- *Aarhus*	52	-0.09	-0.29; 0.10	0.36	0.01	-0.19; 0.22	0.90	
Fish, g/day[b]	186	0.02	0.002; 0.03	0.02	0.02	0.004; 0.03	0.01	
Prawns, g/day	186	0.10	0.03; 0.17	0.008	0.04	-0.03; 0.10	0.01	
Red meat, g/day[c]	186	-0.003	-0.006; 0.001	0.13	-0.002	-0.005; 0.002	0.33	
Poultry, g/day[d]	186	0.003	-0.003; 0.009	0.32	0.004	-0.002; 0.01	0.16	
Tap water, L/day[e]	186	-0.00002	-0.0002; 0.0001	0.74	0.00002	-0.0001; 0.0001	0.79	0.41
All vegetables, incl. juices, g/day	186	-0.0006	-0.001; 0.0001	0.11	-0.0007	-0.002; 0.0004	0.20	
Cruciferous vegetables, g/day	186	-0.001	-0.01; 0.01	0.81	0.004	-0.004; 0.01	0.32	
Leafy vegetables, g/day	186	-0.002	-0.01; 0.01	0.56	-0.0009	-0.01; 0.008	0.85	
All fruits, incl. juices, g/day	186	-0.001	-0.001; 0.0001	0.07	-0.0004	-0.001; 0.0002	0.16	
Potatoes, g/day	186	-0.001	-0.002; -0.0002	0.02	-0.0004	-0.001; 0.0006	0.42	
Rice, g/day	186	0.002	-0.004; 0.008	0.55	0.002	-0.004; 0.007	0.56	
Cereals, g/day	186	-0.0001	-0.002; 0.001	0.84	0.0003	-0.001; 0.002	0.64	
Dairy products, g/day[f]	186	-0.0004	-0.001; -0.0001	0.002	-0.0002	-0.0005; 0.00003	0.08	
Arsenic in drinking-water, µg/L	186	-0.01	-0.06; 0.03	0.60	0.01	-0.04; 0.05	0.67	

Creatinine	186	0.55	0.41; 0.83	<0.0001	0.50	0.36; 0.65	<0.0001
Case status							
- *Case*	97	Ref	-	0.06	Ref	-	0.06
- *Non-case*	89	-0.17	-0.34; 0.004		-0.14	-0.30; 0.009	

[a] Among drinkers
[b] Sum of fresh and processed fish.
[c] Beef, veal, pork and lamb (including processed meat and offal).
[d] Chicken and turkey.
[e] Sum of tap water, coffee, tea, and fruit syrup diluted with tap water.
[f] Milk, cheese, cream, yogurt, ice cream and other cultured milk products.
* Later development of breast cancer (case status) as well as urinary creatinine concentration included as adjustment factors.

Table s2. Association between log_{10}-transformed urinary arsenic levels and baseline study characteristics. Results of forward regression model among women in the lowest quartile of fish-consumption (<= 23.9 g/day) only.

	**Difference (ng/mL)**	**P value**	**Adjusted R^2**
**Study variables**			
Intercept	0.68	-	0
Creatinine	0.51	< 0.0001	0.2571
Alcohol intake (g/day)[a]	0.005	0.002	0.2899
Fish, g/day[b]	0.02	0.01	0.3094
All fruits, incl. juices, g/day	-0.0006	0.01	0.3286
Dairy products, g/day[c]	-0.0002	0.06	0.3379
Case status	-0.12	0.12	0.3431
Potatoes, g/day	-0.0006	0.20	0.3457

[a] Among drinkers
[b] Sum of fresh and processed fish.
[c] Milk, cheese, cream, yogurt, ice cream and other cultured milk products.

Additional details on urinary arsenic analysis

Analysis of urinary arsenic was performed using methods that have been described in a previous reference [1]. Urine samples were prepared for analysis by acid digestion with a mixture of nitric and hydrochloric acid and hydrogen peroxide. A 0.500 mL aliquot of urine was pipetted into a sample preparation vessel and mixed with 0.100 mL of high-purity concentrated nitric acid (Optima purity; Fisher Scientific, Hampton, NH), 0.050 mL high purity concentrated hydrochloric acid (Optima purity; Fisher Scientific), and 0.100 mL of high purity 30% hydrogen peroxide (Suprapur purity; EMD Millipore, Burlington, MA). Samples were then heated on a graphite heating block at a temperature of 90 °C for 30 minutes and allowed to cool to room temperature before spiking with an internal standard mix to a final target concentration of 10 ng/mL and dilution with deionized water to final volume.

An X-series II quadrupole ICP-MS (Thermo, Waltham, MA) was used for determination of arsenic in urine along with a suite of additional metals (antimony, barium, beryllium, cadmium, cesium, cobalt, lead, molybdenum, platinum, thallium, tin, tungsten, uranium, and zinc). Arsenic, cadmium, cobalt, zinc, molybdenum, and tin were all monitored in collision cell mode with a 10% H_2/He gas mixture to minimize the signal contribution arising from polyatomic interferences. This is particularly important for arsenic, as the primary interferent for As is ArCl, which is prevalent in biofluid samples such as urine. Prior to analysis on each analytical day, the instrument response and interference removal in both normal and CCT mode were optimized by tuning the instrument settings in a standard solution containing 10 ppb B, In, Ce, and U. A table showing general instrument settings for analysis is provided below.

Table S3. ICP-MS Parameters.

Instrument	Thermo X-Series 2 ICP-MS
Software	PlasmaLab ICP-MS Software, Version 2.5.3.280
Collision Cell Gas	10% H_2/He
Nebulizer	MicroMist 0.20 mL/min (Glass Expansion, Pocasset, MA)
Spray Chamber	Glass Impact bead
Injector	1.8 mm, quartz
Cones	Ni

Elements were calibrated by analysis of acid-matrix matched standards over the concentration range of 0.333-33.3 ng element/mL sample immediately following tuning. Instrument performance was monitored on a continuing basis by regular reanalysis of a mid-level calibration curve after every

10 samples. Method accuracy was monitored by analysis of spiked method blank samples and the National Institute of Standards and Technology (NIST, Gaithersburg, MD) standard reference material (SRM) 2668 – Toxic Elements in Frozen Human Urine.

The limit of detection (LOD) for arsenic was calculated on each analytical day as the standard deviation of measured arsenic concentration in quality control blanks, multiplied by the student's t-value for the appropriate degrees of freedom at 99% confidence interval, and ranged from 0.51-3.30 ng As/mL urine, with an average of 0.988 ng As/mL urine. Batch-specific limits of quantitation (LOQ) were conservatively defined as the lowest acceptable calibration standard on each analysis day and averaged 1.33 ng/mL. Background elemental concentration arising from sample preparation and instrumental conditions was monitored by preparation and analysis of acid-matrix matched method blank samples, which ranged from below the limit of detection to 2.8 ng As/mL urine and exhibited an average concentration below the limit of detection. Average recovery of National Institute of Standards and Technology (NIST, Gaithersburg, MD) standard reference material (SRM 2668; Toxic Elements in Frozen Human Urine) aliquots was 91.1% across all batches, with a median recovery of 86.8% and a range of 74.2-120%.

1. Eriksen KT, McElroy JA, Harrington JM, Levine KE, Pedersen C, Sorensen M, Tjonneland A, Meliker JR, Raaschou-Nielsen O: **Urinary Cadmium and Breast Cancer: A Prospective Danish Cohort Study**. *Journal of the National Cancer Institute* 2017, **109**(2).
